# Antxr1, Which is a Target of Runx2, Regulates Chondrocyte Proliferation and Apoptosis

**DOI:** 10.3390/ijms21072425

**Published:** 2020-03-31

**Authors:** Qing Jiang, Xin Qin, Carolina Andrea Yoshida, Hisato Komori, Kei Yamana, Shinsuke Ohba, Hironori Hojo, Brad St. Croix, Viviane K. S. Kawata-Matsuura, Toshihisa Komori

**Affiliations:** 1 Basic and Translational Research Center for Hard Tissue Disease, Nagasaki University Graduate School of Biomedical Sciences, Nagasaki 852-8588, Japanvivianekawata@gmail.com (V.K.S.K.-M.); 2 Teijin Institute for Bio-Medical Research, Teijin Limited, Tokyo 100-8585, Japan; 3 Department of Cell Biology, Nagasaki University Graduate School of Biomedical Sciences, Nagasaki 852-8588, Japan; 4 Department of Bioengineering, the University of Tokyo Graduate School of Engineering, Tokyo 113-0033, Japan; 5 Tumor Angiogenesis Unit, National Cancer Institute (NCI), National Institutes of Health (NIH), Frederick, MD 21702, USA

**Keywords:** Antxr1, Runx2, GAPO syndrome, chondrocyte proliferation, apoptosis

## Abstract

Antxr1/Tem8 is highly expressed in tumor endothelial cells and is a receptor for anthrax toxin. Mutation of *Antxr1* causes GAPO syndrome, which is characterized by growth retardation, alopecia, pseudo-anodontia, and optic atrophy. However, the mechanism underlying the growth retardation remains to be clarified. Runx2 is essential for osteoblast differentiation and chondrocyte maturation and regulates chondrocyte proliferation through *Ihh* induction. In the search of Runx2 target genes in chondrocytes, we found that *Antxr1* expression is upregulated by Runx2. *Antxr1* was highly expressed in cartilaginous tissues and was directly regulated by Runx2. In skeletal development, the process of endochondral ossification proceeded similarly in wild-type and *Antxr1*^–/–^ mice. However, the limbs of *Antxr1*^–/–^ mice were shorter than those of wild-type mice from embryonic day 16.5 due to the reduced chondrocyte proliferation. Chondrocyte-specific *Antxr1* transgenic mice exhibited shortened limbs, although the process of endochondral ossification proceeded as in wild-type mice. BrdU-uptake and apoptosis were both increased in chondrocytes, and the apoptosis-high regions were mineralized. These findings indicated that Antxr1, of which the expression is regulated by Runx2, plays an important role in chondrocyte proliferation and that overexpression of *Antxr1* causes chondrocyte apoptosis accompanied by matrix mineralization.

## 1. Introduction

Anthrax toxin receptor 1 (Antxr1)/tumor endothelial marker 8 (Tem8) was discovered in the screening of proteins expressed in tumor endothelium at an increased level and identified as a receptor for protective antigen (PA) of anthrax toxin [1,2]. Mutation of *Antxr1* results in GAPO syndrome, which is an autosomal recessive disorder and characterized by growth retardation, alopecia, pseudo-anodontia, and progressive visual impairment. Histologically, it is also characterized by an abnormal accumulation of extracellular matrix [3,4]. Antxr1 in the tumor endothelium interacts with the C-terminal C5 domain of collagen α3(VI) [5]. Antxr1 functions as an adhesion molecule for cell spreading, interacts with PA and collagen I through its extracellular domain, and interacts with actin cytoskeleton through its intracellular domain [6]. *Antxr1*^–/–^ mice exhibit dwarfism; excess extracellular matrix in ovaries, uterus, skin, and periodontal ligament of the incisors; and the accumulation of collagen I and VI [7,8]. Moreover, there is increased proliferation of endothelial cells, in which the vascular endothelial growth factor receptor (VEGFR) signaling pathway is activated, and the excess extracellular matrix is caused by increased synthesis by fibroblastic cells and reduced degradation by Mmp2 in the skin of *Antxr1*^–/–^ mice [8]. Although *Antxr1*^–/–^ mice exhibit dwarfism, the function of Antxr1 in skeletal development remains to be clarified.

Runx2 is an essential transcription factor for osteoblast differentiation and chondrocyte maturation [9]. Runx2 induces the proliferation of osteoblast progenitors through the induction of *Fgfr2* and *Fgfr3* and induces the proliferation of osteoblast progenitors and their commitment into osteoblast lineage cells through reciprocal regulation with hedgehog, Wnt, and Phtlh signaling pathways [10,11]. Runx2 also regulates the expression of Sp7, an essential transcription factor for osteoblast differentiation [12], and induces the differentiation of preosteoblasts into immature osteoblasts [13,14]. After the differentiation to immature osteoblasts, Runx2 induces the expression of bone matrix protein genes, including *Spp1*, *Ibsp*, and *Bglap2*, which regulates the mineralization and alignment of apatite crystals [14,15]. The growth plate is composed of resting, proliferating, prehypertrophic, hypertrophic, and terminal hypertrophic chondrocyte layers. *Col2a1* is expressed in resting, proliferating, and prehypertrophic chondrocyte layers; *Ihh* is expressed in the prehypertrophic chondrocyte layer; *Col10a1* is expressed in the hypertrophic chondrocyte layer; and *Spp1* is expressed in the terminal hypertrophic chondrocyte layer and in osteoblasts [16]. *Runx2* is weakly expressed in resting and proliferating chondrocytes and is upregulated in prehypertrophic chondrocytes, and its expression is maintained in hypertrophic and terminal hypertrophic chondrocytes [16,17]. Runx2 induces maturation of prehypertrophic chondrocytes into hypertrophic chondrocytes, and Runx3 is also partly involved in this maturation [18,19,20,21]. Runx2 regulates chondrocyte proliferation through the induction of *Ihh* expression in the prehypertrophic chondrocytes, which directly induces chondrocyte proliferation in the proliferating chondrocyte layer [21,22]. Ihh induces Pthlh, which inhibits chondrocyte maturation at least partly through the inhibition of *Runx2* expression, forming a negative feedback loop for chondrocyte maturation [23,24].

We found that *Antxr1* expression is upregulated by Runx2 in the screening of Runx2 target genes in chondrocytes. We report that *Antxr1* expression is directly regulated by Runx2, that chondrocyte proliferation is reduced in *Antxr1*^–/–^ mice, and that the overexpression of *Antxr1* in chondrocytes increases DNA replication and apoptosis.

## 2. Results

### 2.1. Antxr1 Expression Is Directly Regulated by Runx2

**Figure 1:**
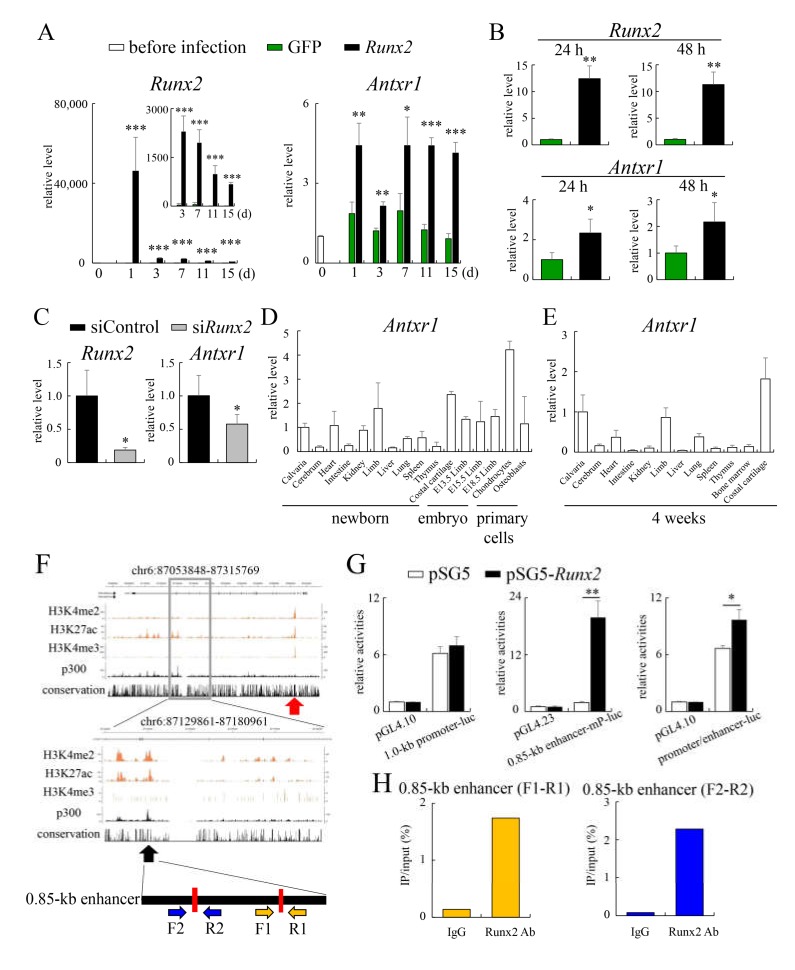
Real-time RT-PCR analysis, reporter assay, and ChIP: (**A**,**B**) Induction of *Antxr1* by Runx2. Primary chondrocytes from *Runx2*^–/–^ embryos at E18.5 (**A**) or wild-type embryos at E15.5 (**B**) were infected with an adenovirus expressing green fluorescent protein (GFP) or *Runx2*-GFP, and *Runx2* and *Antxr1* expressions were analyzed by real-time RT-PCR. The values before infection (**A**) and those in GFP-expressing cells (**B**) were defined as 1, and relative levels are shown. *n* = 3–4. ∗Versus GFP. * *p* < 0.05, ** *p* < 0.01, *** *p* < 0.001. (**C**) Reduction of *Antxr1* by si*Runx2*: Primary chondrocytes were infected with siControl or si*Runx2*. RNA was extracted after 48 h, and *Runx2* and *Antxr1* expressions were analyzed by real-time RT-PCR. The values of siControl were defined as 1, and relative levels are shown. *n* = 4. * Versus siControl. * *p* < 0.05. (**D**,**E**) Real-time RT-PCR analysis of *Antxr1* expression using RNA from the tissues at the newborn stage; limb skeletons at E13.5, E15.5, and E18.5; and primary cells (**D**) and the tissues at 4 weeks of age (**E**) from wild-type mice. The values in calvaria were defined as 1, and relative levels are shown. The samples were prepared from 3–4 mice at each age. (**F**) ChIP-seq enrichment profiles of H3k4me2, H3K27ac, H3K4me3, and p300 in mouse rib chondrocytes at the *Antxr1* gene locus: The scale indicates the intensity of enrichment. Red arrow indicates the peaks around exon 1. The locations of Runx2-binding motifs (red bars) and the primer sets for ChIP are shown on the bottom. (**G**) Reporter assays: Relative luciferase activity of 1.0-kb promoter-luc, 0.85-kb enhancer-minimal promoter (mP)-luc, and 1.0-kb promoter/0.85-kb enhancer-luc in ATDC5 cells transfected with the empty vector (pSG5) or Runx2 expression vector (pSG5-*Runx2*). * *p* < 0.05, ** *p* < 0.01. *n* = 4. (**H**) ChIP assays: DNA before immunoprecipitation (input) and after immunoprecipitation (IP) with anti-Runx2 antibody or rabbit IgG was amplified by real-time PCR using the primers shown in Figure 1F. The percentages of the amplified DNA from IP against that from the input are shown.

To identify Runx2 target genes in chondrocytes, we introduced *Runx2*-expressing adenovirus or green fluorescent protein (GFP)-expressing adenovirus into *Runx2*^–/–^ primary chondrocytes and examined the differentially expressed genes by microarray [23,24]. *Antxr1* expression was upregulated 3.7 times by *Runx2*-expressing adenovirus infection in the microarray analysis. We introduced *Runx2*- or GFP-expressing adenovirus into *Runx2*^–/–^ primary chondrocytes or wild-type primary chondrocytes and examined *Antxr1* expression by real-time RT-PCR (Figure 1A,B). *Antxr1* expression was upregulated by *Runx2* introduction. Furthermore, *Antxr1* expression was reduced by *Runx2* siRNA introduction (Figure 1C). *Antxr1* was strongly expressed in the calvaria, heart, kidneys, limbs, and costal cartilage at the newborn stage and in the calvaria, limbs, and costal cartilage at 4 weeks of age (Figure 1D,E). In both developmental stages, *Antxr1* was most highly expressed in costal cartilage. Furthermore, *Antxr1* was highly expressed in limb skeletons during the embryonic stage and in primary chondrocytes, which originated from limb skeletons at E15.5 (Figure 1D). Thus, we selected *Antxr1* as a Runx2 target gene candidate in chondrocytes.

To investigate whether *Antxr1* expression is directly regulated by Runx2, we examined Runx2-binding regulatory regions in the *Antxr1* region by reanalyzing our previously published chromatin immunoprecipitation (ChIP)-seq data of H3K4me2, H3K4me3, H3K27ac, and p300 in mouse primary rib chondrocytes [25]. There were peaks of H3K4me2, H3K4me3, and H3K27ac around exon 1 (Figure 1F). In a reporter assay using a 1.0-kb DNA fragment covering the regions of the three peaks and the conserved sequence among vertebrates around exon 1, the 1.0-kb DNA fragment had strong promoter activity. However, Runx2 failed to enhance the reporter activity, suggesting that it induces *Antxr1* expression through enhancers (Figure 1F,G). *Antxr1* is composed of 18 exons. By searching for genomic elements marked by tissue-specific active enhancer signatures (H3K4me2^high^/H3K4me3^low^/H3K27ac^+^/p300^+^) in the ChIP-seq data, we found one enhancer candidate in the 14th intron (Figure 1F). There were two Runx2-binding motifs in this region. The 0.85-kb DNA fragment containing the enhancer candidate was inserted into the luciferase vector containing a minimal promoter. Runx2 strongly increased the reporter activity (Figure 1G). Runx2 also enhanced the reporter activity of 1.0-kb promoter-luciferase-0.85-kb enhancer construct (Figure 1G). In addition, ChIP analysis using Runx2 antibody demonstrated that Runx2 binds to both Runx2-binding motifs in the 0.85-kb region (Figure 1H). This suggested that Runx2 regulates *Antxr1* expression mainly through the 0.85-kb enhancer.

### 2.2. The Process of Endochondral Ossification Proceeded Normally in Antxr1^–/–^ Mice, but Their Femurs and Tibiae Were Shorter than Those of Wild-Type Mice due to Reduced Chondrocyte Proliferation

**Figure 2:**
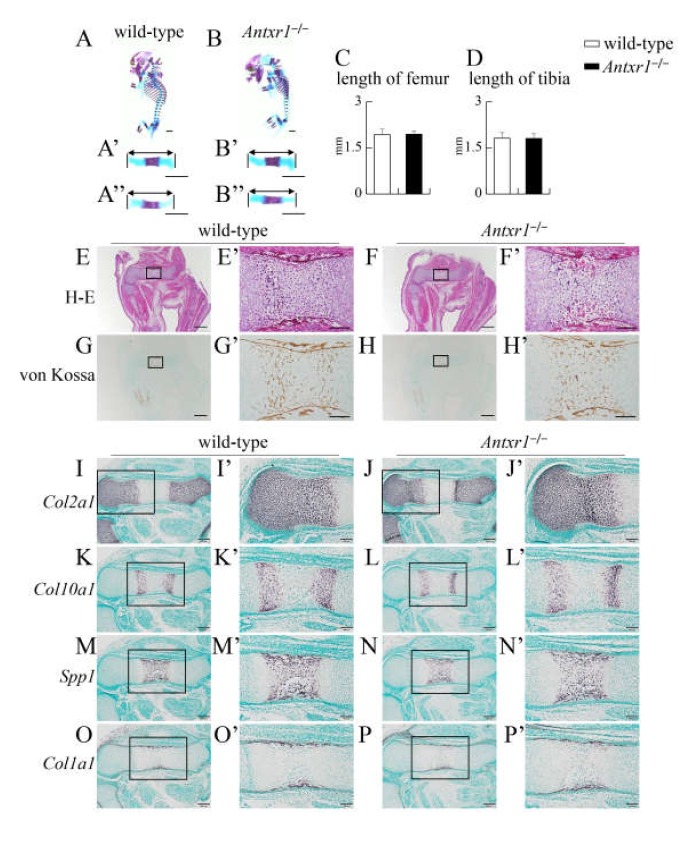
Skeletal system and histological analyses of wild-type and *Antxr1*^–/–^ embryos at E15.5: (**A**,**B**) Lateral view of the whole skeletons (**A**,**B**) and the enlarged views of femurs (**A’**,**B’**) and tibiae (**A’’**,**B’’**) of wild-type (**A**) and *Antxr1*^–/–^ (**B**) embryos. The lengths of femurs (**C**) and tibiae (**D**): The lengths of both sides of femurs and tibiae were measured using the skeletal preparations of 4 wild-type and 4 *Antxr1*^–/–^ embryos. (**E**–**H**) Histological analyses by H-E (**E**,**F**) and von Kossa (**G**,**H**) staining using femoral sections from wild-type (**E**,**G**) and *Antxr1*^–/–^ (**F**,**H**) embryos. (**I**–**P**) In situ hybridization using *Col2a1* (I,J), *Col10a1* (K,L), *Spp1* (M,N), and *Col1a1* (O,P) probes. The boxed regions in **E**–**P** are magnified in **E’**–**P’**, respectively. Scale bars: 1 mm (**A**,**B**), 500 µm (**E**–**H**), 200 µm (**I**–**P**), and 100 µm (**E’**–**P’**). The number of embryos analyzed: H-E staining, wild-type: 9, *Antxr1*^–/–^: 5; von Kossa staining, wild-type: 7, *Antxr1*^–/–^: 3; in situ hybridization, wild-type: 2, *Antxr1*^–/–^: 3. Similar results were obtained and the representative data are shown.

**Figure 3:**
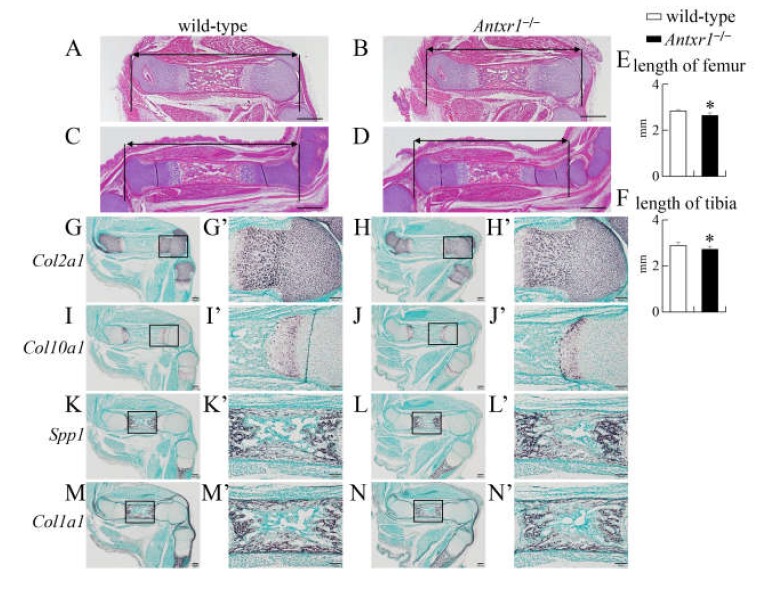
Histological analyses of wild-type and *Antxr1*^–/–^ embryos at E16.5: (**A**–**D**) H-E staining of femurs (**A**,**B**) and tibiae (**C**,**D**) in wild-type (**A**,**C**) and *Antxr1*^–/–^ (**B**,**D**) embryos. (**E**,**F**) The lengths of femurs (**E**) and tibiae (**F**): The lengths of femurs and tibiae were measured using histological sections of 6 wild-type and 4 *Antxr1*^–/–^ embryos, as shown in **A**–**D**. Versus wild-type embryos, * *p* < 0.05. (**G**–**N**) In situ hybridization using *Col2a1* (**G**,**H**), *Col10a1* (**I**,**J**), *Spp1* (**K**,**L**), and *Col1a1* (**M**,**N**) probes. The boxed regions in **G**–**N** are magnified in **G’**–**N’**, respectively. Three embryos of each genotype were analyzed by in situ hybridization and showed similar results. Scale bars: 500 µm (**A**–**D**), 200 µm (**G**–**N**), and 100 µm (**G’**–**N’**).

**Figure 4:**
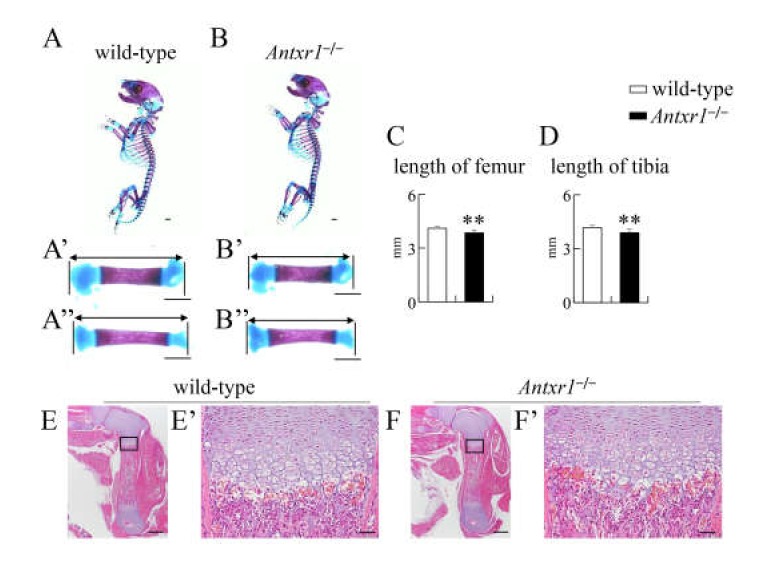
Skeletal system and histological analysis of wild-type and *Antxr1*^–/–^ newborns: (**A**,**B**) Lateral view of the whole skeletons (**A**,**B**) and magnified pictures of femurs (**A’**,**B’**) and tibiae (**A’’**,**B’’**) in wild-type (**A**) and *Antxr1*^–/–^ (**B**) newborns. (**C**,**D**) The lengths of femurs (**C**) and tibiae (**D**): The lengths of both sides of femurs and tibiae were measured using skeletal preparations of 4 wild-type and 3 *Antxr1*^–/–^ newborns, as shown in **A’**,**B’**,**A’’** and **B’’**. Versus wild-type newborns, **p < 0.01. (**E**,**F**) H-E staining using femoral sections from wild-type (**E**) and *Antxr1*^–/–^ (**F**) newborns. The boxed regions in E and F are magnified in E’ and F’, respectively. Scale bars: 1 mm (**A**,**B**), 500 µm (**E**,**F**), and 100 µm (**E’**,**F’**). The number of mice analyzed: H-E staining, wild-type: 2, *Antxr1*^–/–^: 2.

In skeletal preparations at E15.5, mineralization was similar and the lengths of femurs and tibiae were similar between wild-type and *Antxr1*^–/–^ mice (Figure 2A–D). There was also no apparent difference on histological analysis by hematoxylin and eosin (H-E) or von Kossa staining (Figure 2E–H). Based on in situ hybridization using *Col2a1*, *Col10a1*, *Spp1*, and *Col1a1* probes at E15.5 and E16.5, the process of endochondral ossification proceeded similarly in wild-type and *Antxr1*^–/–^ mice (Figure 2I–P and 3G–N). However, the lengths of femurs and tibiae were shorter in *Antxr1*^–/–^ mice than in wild-type mice at E16.5 (Figure 3A–F). Although mineralization in skeletal preparations was similar and histological analysis demonstrated no apparent difference between wild-type and *Antxr1*^–/–^ mice, the lengths of femurs and tibiae in *Antxr1*^–/–^ mice were also shorter than those in wild-type mice at the newborn stage (Figure 4).

**Figure 5:**
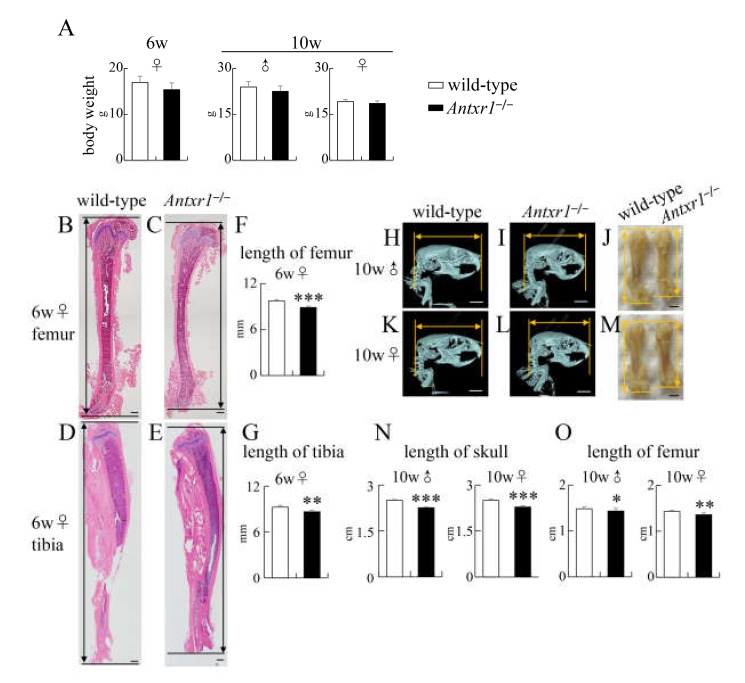
Body weight and histological and micro-CT analyses of wild-type and *Antxr1*^–/–^ mice at 6 weeks and 10 weeks of age: (**A**) Body weights of female mice at 6 weeks of age and of male and female mice at 10 weeks of age. (**B**–**E**) H-E staining of femurs (**B**,**C**) and tibiae (**D**,**E**) in female wild-type (**B**,**D**) and *Antxr1*^–/–^ (**C**,**E**) mice at 6 weeks of age. (**F**,**G**) The lengths of femurs and tibiae at 6 weeks of age were measured using histological sections, as shown in **B**–**E**. (**H**,**I**,**K**,**L**) Lateral view of micro-CT images of skulls of male (**H**) and female (**K**) wild-type mice and of male (**I**) and female (**L**) *Antxr1*^–/–^ mice at 10 weeks of age. (**J**,**M**) The appearance of femurs in male wild-type and *Antxr1*^–/–^ mice (**J**) and of female wild-type and *Antxr1*^–/–^ mice (M) at 10 weeks of age. (**N**) The skull length at 10 weeks of age was measured using micro-CT images, as shown in **H**, **I**, **K**, and **L**. (**O**) The length of femurs at 10 weeks of age was measured, as shown in **J** and **M**. Scale bars: 500 µm (**B**–**E**), 0.5 cm (**H**,**I**,**K**,**L**), and 0.2 cm (**J**,**M**). The number of mice analyzed: body weight at 6 weeks of age, wild-type: 5, *Antxr1*^–/–^: 5; the length of femurs at 6 weeks of age, wild-type: 4, *Antxr1*^–/–^: 5; body weights and the lengths of skulls and femurs in male mice at 10 weeks of age, wild-type: 12, *Antxr1*^–/–^: 7; body weights and the lengths of skulls and femurs in female mice at 10 weeks of age, wild-type: 7, *Antxr1*^–/–^: 9. Versus wild-type mice, * *p* < 0.05, ** *p* < 0.01, *** *p* < 0.001.

The body weight of *Antxr1*^–/–^ mice was similar to that of wild-type mice at 6 weeks of age, but the lengths of femurs and tibiae in *Antxr1*^–/–^ mice were shorter than those in wild-type mice (Figure 5A–G). The body weights of both male and female *Antxr1*^–/–^ mice were also similar to those of wild-type mice at 10 weeks of age, but the skull length in the sagittal plane and femur length in *Antxr1*^–/–^ mice were shorter than those in wild-type mice at 10 weeks of age in both sexes (Figure 5A,H–O). Therefore, the lengths of lower limbs in *Antxr1*^–/–^ mice were consistently shorter than those in wild-type mice after E16.5, irrespective of their similar body weights.

**Figure 6:**
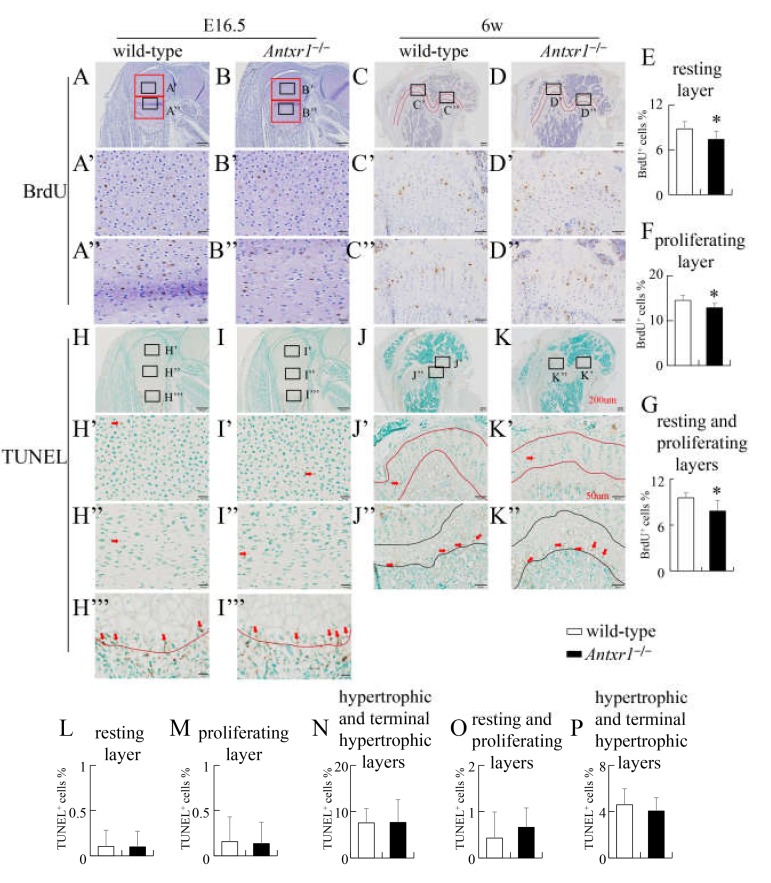
Proliferation and apoptosis of chondrocytes in wild-type and *Antxr1*^–/–^ mice at E16.5 and 6 weeks of age: (**A**–**D**) BrdU staining using femoral sections from wild-type (**A**,**C**) and *Antxr1*^–/–^ (**B**,**D**) mice at E16.5 (**A**,**B**) and 6 weeks of age (**C**,**D**). The boxed regions in **A**–**D** are magnified in **A’**–**D’** and **A’’**–**D’’**. (**E**–**G**) The frequencies of BrdU-positive chondrocytes in the resting (**E**) and proliferating (**F**) layers (red boxes in **A** and **B**) at E16.5 and those in the resting and proliferating layers (the area between two red lines in **C** and **D**) at 6 weeks of age (**G**). The layer with columnar alignment of chondrocytes without hypertrophy was regarded as a proliferating layer, and the epiphyseal part of the proliferating layer was regarded as a resting layer. (**H**–**K**) TUNEL staining using femoral sections from wild-type (**H**,**J**) and *Antxr1*^–/–^ (**I**,**K**) mice at E16.5 (**H**,**I**) and 6 weeks of age (**J**,**K**). The boxed regions in **H**–**K** are magnified in **H’**–**K’**, **H’’**–**K’’**, **H’’’**, and **I’’’**. The red arrows in **H’**–**I’’’** indicate TUNEL-positive chondrocytes. (**L**–**P**) The frequencies of TUNEL-positive chondrocytes in the resting layers (**H’**,**I’**,**L**), proliferating layers (**H’’**,**J’’**,**M**), and hypertrophic and terminal hypertrophic layers (the upper part of the line in **H’’’** and **I’’’**) (**N**) at E16.5 and those in the resting and proliferating layers (the area between the two red lines in **J’** and **K’**) (**O**) and hypertrophic and terminal hypertrophic layers (the area between the two black lines in **J’’** and **K’’**) (**P**) at 6 weeks of age. The layer with chondrocyte hypertrophy was regarded as hypertrophic and terminal hypertrophic layers. Scale bars: 200 µm (**A**–**D**, **H**–**K**), 50 µm (**A’**–**D’**,**A’’**–**D’’**,**H’**–**K’**,**H’’**–**K’’**,**H’’’**,**I’’’**). The number of mice analyzed: BrdU staining at E16.5, wild-type: 7, *Antxr1*^–/–^: 6; BrdU staining at 6 weeks of age, wild-type: 5, *Antxr1*^–/–^: 5; TUNEL staining at E16.5, wild-type: 3, *Antxr1*^–/–^: 3; TUNEL staining at 6 weeks of age, wild-type: 3, *Antxr1*^–/–^: 3. Two sections were counted for each mouse. Versus wild-type mice, *p < 0.05.

To investigate the reason for the shortening of the lower limbs in *Antxr1*^–/–^ mice, we performed 5-bromo-2-deoxyuridine (BrdU)-labeling at E16.5 and 6 weeks of age. The percentages of BrdU-positive chondrocytes in *Antxr1*^–/–^ mice were significantly lower than the respective values in wild-type mice in both resting and proliferating layers at E16.5 (Figure 6A,B,E,F). The percentage of BrdU-positive chondrocytes in the resting and proliferating layers in *Antxr1*^–/–^ mice was also lower than that in wild-type mice at 6 weeks of age (Figure 6C,D,G). We next examined apoptosis by terminal deoxynucleotidyl transferase-mediated dUTP nick end labeling (TUNEL) staining. There were no differences in the percentages of TUNEL-positive cells in the resting layer, proliferating layer, and hypertrophic and terminal hypertrophic layers between wild-type and *Antxr1*^–/–^ mice at E16.5 or 6 weeks of age (Figure 6H–P).

### 2.3. The Process of Endochondral Ossification Proceeded Normally, but Ectopic Mineralization Was Observed in Chondrocyte-Specific Antxr1 Transgenic (tg) Mice

**Figure 7:**
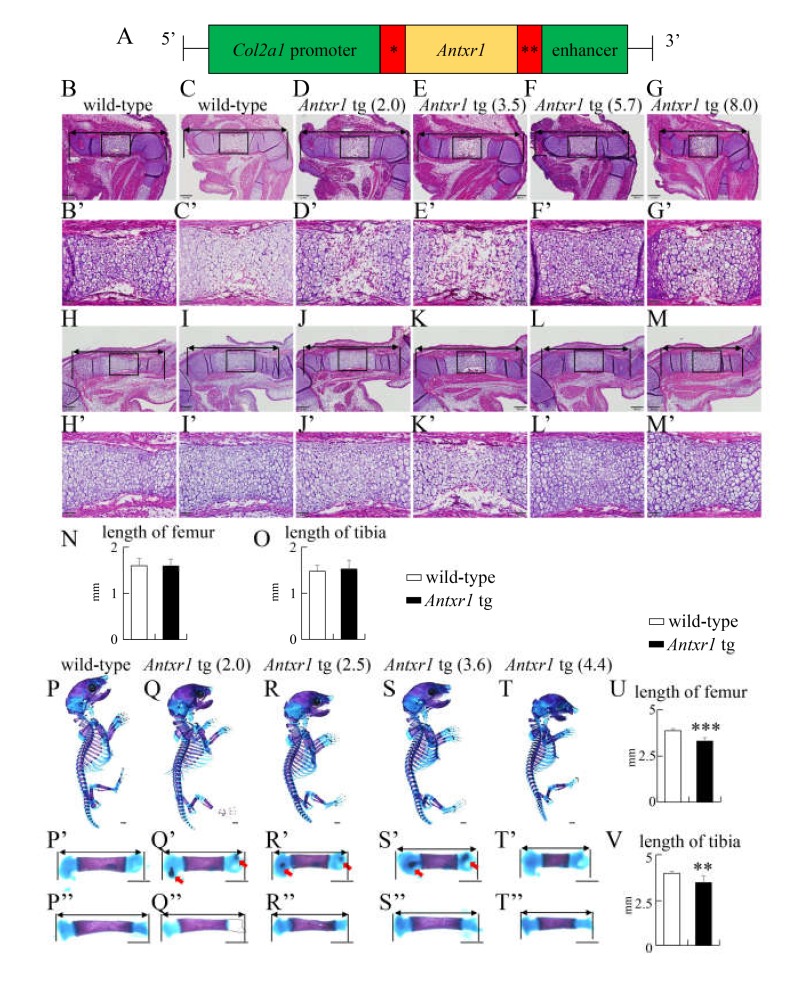
Generation of *Col2a1*-*Antxr1* tg mice and histological and skeletal system analyses of wild-type and *Antxr1* tg embryos at E15.5 and E18.5: (**A**) Diagram of the DNA construct used to generate tg mice that express *Antxr1* under the control of the mouse *Col2a1* promoter-enhancer. * Intron from rabbit β globin gene, **Polyadenylation signal from SV40. (**B–M**) H-E staining of femurs (**B**–**G**) and tibiae (**H**–**M**) from wild-type (**B**,**C**,**H**,**I**) and *Antxr1* tg (**D**–**G**, **J**–**M**) F_0_ embryos at E15.5. The expression level of *Antxr1* in wild-type embryos was set as 1, and the relative levels in tg embryos are shown in parenthesis. The boxed regions in B–M are magnified in B’–M’, respectively. (**N**,**O**) The lengths of femurs (**N**) and tibiae (**O**) at E15.5 were measured using the sections from 5 wild-type and 4 *Antxr1* tg embryos at E15.5, as shown in B–M. (**P**–**T**) Lateral view of the whole skeletons of wild-type (**P**) and *Antxr1* tg (**Q**–**T**) F_0_ embryos at E18.5: The expression levels are shown in parentheses. The femurs and tibiae were magnified in **P’**–**T’** and **P’’**–**T’’**, respectively. The arrows in **Q’**, **R’**, and **S’** show ectopic mineralization in the cartilage. (**U**,**V**) The lengths of femurs (**U**) and tibias (**V**) at E18.5 were measured using skeletal preparations, as shown in **P’**–**T’** and **P’’**–**T’’**. Scale bars: 200 µm (**B**–**M**), 50 µm (**B’**–**M’**), 1 mm (**P**–**T**,**P’**–**T’**,**P’’**–**T’’**). The number of embryos analyzed: H-E staining at E15.5, wild-type: 5, *Antxr1* tg: 4; skeletal system at E18.5, wild-type: 9, *Antxr1* tg: 4. Versus wild-type embryos, ** *p* < 0.01, *** *p* < 0.001.

**Figure 8:**
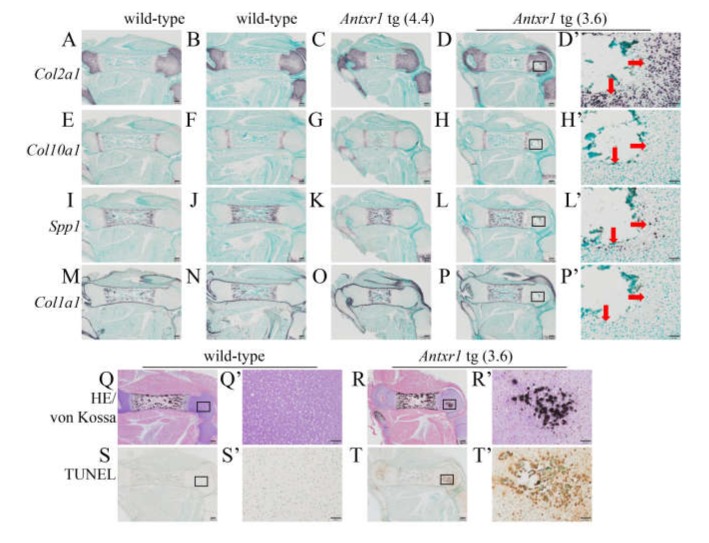
Histological analyses of wild-type and *Antxr1* tg embryos at E18.5: (**A**–**P**) In situ hybridization using femoral sections from wild-type (**A**,**B**,**E**,**F**,**I**,**J**,**M**,**N**) and *Antxr1* tg (**C**,**D**,**G**,**H**,**K**,**L**,**O**,**P**) F_0_ embryos. *Col2a1* (**A**–**D**), *Col10a1* (**E**–**H**), *Spp1* (**I**–**L**), and *Col1a1* (**M**–**P**) anti-sense probes were used. The boxed regions in **D**, **H**, **L**, and **P** are magnified in **D’**, **H’**, **L’**, and **P’**, respectively. The arrows in **D’**, **H’**, **L’**, and **P’** show the location of *Spp1*-positive cells. (**Q**–**T**) Double-staining with H-E and von Kossa (**Q**,**R**) and TUNEL staining (**S**,**T**) of wild-type (**Q**,**S**) and *Antxr1* tg (**R**,**T**) F_0_ embryos: The boxed regions in **Q**–**T** are magnified in **Q’**–**T’**, respectively. Scale bars: 200 µm (**A**–**T**), 50 µm (**D’**,**H’**,**L’**,**P’**–**T’**). The expression levels in tg embryos are shown in parentheses. The number of embryos analyzed in situ hybridization, wild-type: 3, *Antxr1* tg: 2; double staining of H-E and von Kossa, wild-type: 9, *Antxr1* tg; 4; TUNEL staining, wild-type: 4, *Antxr1* tg: 4. Similar results were obtained and the representative data are shown.

To further assess the functions of Antxr1 in chondrocytes, we generated *Antxr1* tg mice using the *Col2a1* promoter and enhancer and analyzed the F_0_ mice at E15.5 and E18.5 because some tg mice died after birth (Figure 7A). We obtained 67 and 199 F_0_ embryos at E15.5 and E18.5, respectively, and 14 embryos at E15.5 and 30 embryos at E18.5 were *Antxr1* tg. Chondrocyte maturation in *Antxr1* tg mice, in which *Antxr1* expression was 2–8 times higher than endogenous *Antxr1* expression, proceeded similarly to that in wild-type mice at E15.5, and the lengths of femurs and tibiae were similar between wild-type and *Antxr1* tg mice (Figure 7B–O, 7B’–M’). In skeletal preparations at E18.5, the limbs were shorter and the rib cage was smaller in *Antxr1* tg mice, in which the *Antxr1* expression was 2–4 times higher than endogenous *Antxr1* expression; however, mineralization was similar between wild-type and *Antxr1* tg mice, except ectopic mineralization in femurs (Figure 7P–T,P’–T’,P’’–T’’). The lengths of femurs and tibiae in *Antxr1* tg mice were shorter than those in wild-type mice (Figure 7U,V). The opposite lower limbs of the same mice were used to perform in situ hybridization. The expression patterns of *Col2a1*, *Col10a1*, *Spp1*, and *Col1a1* were similar between wild-type and *Antxr1* tg mice (Figure 8A–P). This suggested that the processes of endochondral ossification proceeded similarly in wild-type and *Antxr1* tg mice. However, *Antxr1* tg mice with 18 times higher *Antxr1* expression exhibited dwarfism, markedly shortened limbs, small rib cage, delayed mineralization, and ectopic mineralization in tibia (Supplementary Figure S1A–F). Ectopic mineralization was also observed in vertebra (Supplementary Figure S1G–J).

### 2.4. Ectopic Mineralization Was Caused by Apoptosis Without Chondrocyte Maturation

**Figure 9:**
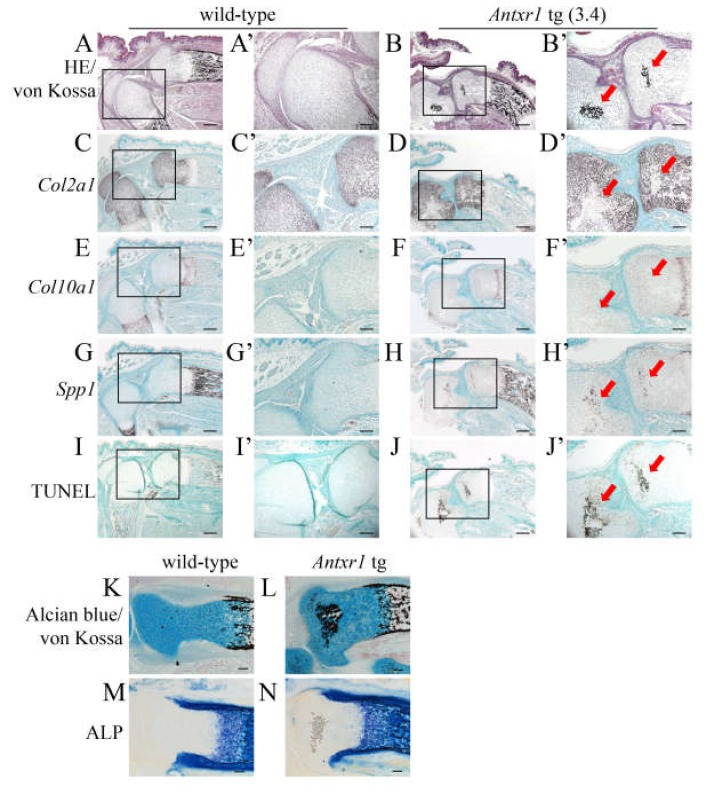
Chondrocyte maturation in the mineralized and apoptosis-enriched regions at E18.5: (**A**–**J**) Double-staining with H-E and von Kossa (**A**,**B**), in situ hybridization (**C**–**H**), and TUNEL staining (**I**,**J**) using hind limbs of wild-type (**A**,**C**,**E**,**G**,**I**) and *Antxr1* tg (**B**,**D**,**F**,**H**,**J**) F_0_ embryos. In situ hybridization was performed using *Col2a1* (**C**,**D**), *Col10a1* (**E**,**F**), and *Spp1* (**G**,**H**) anti-sense probes. The boxed regions in **A**–**J** are magnified in **A’**–**J’**, respectively. The expression level in the tg embryo is shown in parenthesis. The arrows in **B’**, **D’**, **F’**, **H’**, and **J’** indicate the regions with ectopic mineralization. (**K**–**N**) Double-staining with Alcian blue and von Kossa (**K**,**L**) and Alkaline phosphatase (ALP) staining (**M**, **N**) using frozen sections of tibiae of wild-type (**K**,**M**) and *Antxr1* tg (**L**,**N**) embryos. Scale bars: 200 µm (**A**–**J**) and 100 µm (**A’**–**J’**,**K**–**N**).

Von Kossa staining and TUNEL staining showed that the region with mineralization in the resting layer of the femur in serial sections from *Antxr1* tg mice (*Antxr1* tg (3.6)), in which *Antxr1* expression was 3.6 times higher than in wild-type mice, was enriched with TUNEL-positive cells (Figure 8Q–T, Q’–T’), although the mineralized region was detached from the sections used for in situ hybridization (Figure 8D,H,L,P,D’,H’,L’,P’). In the growth plate in wild-type mice, the matrix of terminal hypertrophic chondrocytes is mineralized, which is considered to be caused by the apoptosis of terminal hypertrophic chondrocytes due to their increased intracellular phosphate ions [26,27,28]. To investigate whether the mineralization and apoptosis occurred through chondrocyte maturation, limb bones with ectopic mineralization were carefully examined. The cells surrounding the mineralized region were not hypertrophic and expressed *Col2a1* and/or *Spp1* but not *Col10a1* or *Col1a1* (Figure 8D’,H’,L’,P’,R’). We also examined the lower limb sections from another *Antxr1* tg mouse (*Antxr1* tg (3.4)). The mineralized regions were enriched with TUNEL-positive cells (Figure 9B’,J’). The mineralized regions were negative for *Col2a1* and *Col10a1*, and the surrounding cells were *Col2a1*-positive and/or *Spp1*-positive but *Col10a1*-negative (Figure 9B’,D’,F’,H’). Furthermore, double staining with Alcian blue, which stains proteoglycan in the cartilage, and von Kossa demonstrated mineralization in the Alcian blue-stained area (Figure 9K,L). Alkaline phosphatase (ALP) staining using the serial sections revealed that ALP activity was positive in the terminal hypertrophic chondrocytes and osteoblasts in both wild-type and *Antxr1* tg mice, whereas the ectopically mineralized region in *Antxr1* tg mice was negative for ALP activity (Figure 9M,N). Moreover, neither blood vessels nor hematopoietic cells were observed in the ectopically mineralized regions in histological analyses (Figure 8 and Figure 9, and data not shown). These findings indicate that mineralization and apoptosis occurred in *Antxr1* tg mice in the absence of chondrocyte maturation, vascular invasion, and transdifferentiation of chondrocytes into osteoblasts.

### 2.5. BrdU Uptake Was Increased in Both Resting and Proliferating Layers, and TUNEL-Positive Cells Were Markedly Increased in Both Layers but More Prominently in the Resting Layer in Antxr1 tg Mice

**Figure 10:**
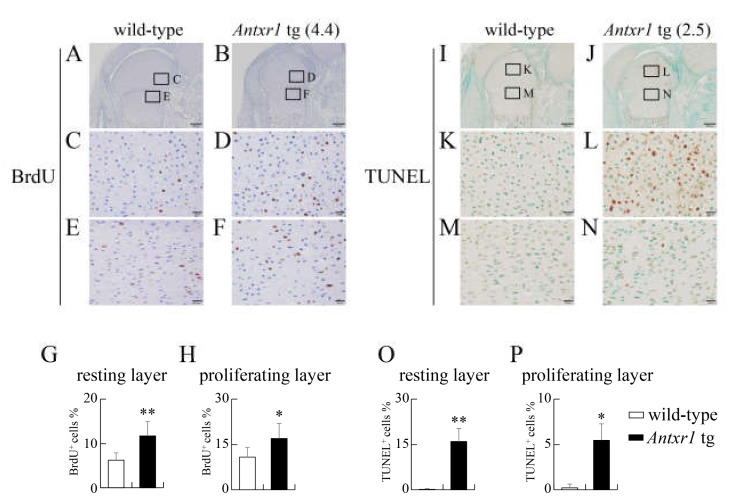
Proliferation and apoptosis in femurs of wild-type and *Antxr1* tg embryos at E18.5: (**A**–**H**) BrdU staining (**A**–**F**) and the frequencies of BrdU-positive chondrocytes in the resting (**A**–**D**,**G**) and proliferating (**A**,**B**,**E**,**F**,**H**) layers of wild-type (**A**,**C**,**E**) and *Antxr1* tg (**B**,**D**,**F**) F_0_ embryos. (**I**–**P**) TUNEL staining (**I**–**N**) and the frequencies of TUNEL-positive chondrocytes in the resting (**I**,**J**,**K**,**L**,**O**) and proliferating (**I**,**J**,**M**,**N**,**P**) layers in wild-type (**I**,**K**,**M**) and *Antxr1* tg (**J**,**L**,**N**) F_0_ embryos at E18.5. The boxed regions in **A**, **B**, **I**, and **J** are magnified in **C** and **E**, in **D** and **F**, in **K** and **M**, and in **L** and **N**, respectively. The expression levels in tg embryos are shown in parentheses. Scale bars: 200 µm (**A**,**B**,**I**,**J**) and 20 µm (**C**–**F**, **K**–**N**). The number of mice analyzed: BrdU staining, wild-type: 6, *Antxr1* tg: 4; TUNEL staining, wild-type: 4, *Antxr1* tg: 4. The expression levels in tg embryos analyzed are shown in Figure 7Q–T. Versus wild-type, *p < 0.05, ** *p* < 0.01.

The limb length was shorter in *Antxr1* tg mice than in wild-type mice, but the process of endochondral ossification proceeded similarly in wild-type and *Antxr1* tg mice. Therefore, we examined chondrocyte proliferation by BrdU labelling and apoptosis by TUNEL staining at E18.5. BrdU-positive cells were increased in resting and proliferating layers of femurs in *Antxr1* tg mice compared with wild-type mice (Figure 10A–H). Furthermore, TUNEL-positive cells were markedly increased in both resting and proliferating layers of femurs in *Antxr1* tg mice compared with wild-type mice (Figure 10I–P). However, the degree of increase was more marked in the resting layer than in the proliferating layer in *Antxr1* tg mice (Figure 10I–P).

## 3. Discussion

The mechanism of abnormal accumulation of extracellular matrix in GAPO syndrome, which is caused by *Antxr1* mutation, has been well studied [29]. Although growth retardation is a common feature of GAPO syndrome, the mechanism remains to be clarified. The shortening of lower limbs was evident in *Antxr1*^–/–^ mice at E16.5 and remained thereafter. BrdU uptake was reduced in *Antxr1*^–/–^ mice at E16.5 and 6 weeks of age. As there was no abnormal accumulation of extracellular matrix in limbs during the embryonic stage and the process of endochondral ossification proceeded normally in *Antxr1*^–/–^ mice, the growth retardation in *Antxr1*^–/–^ mice was mainly caused by reduced chondrocyte proliferation. As *Antxr1* expression was directly regulated by Runx2, Runx2 regulates chondrocyte proliferation not only through the induction of *Ihh* expression [21] but also through the induction of *Antxr1* expression. This is the first report demonstrating that Antxr1 positively regulates cell proliferation in physiological conditions.

Antxr1 inhibits the Vegfa signaling pathway in endothelial cells, and *Antxr1*^–/–^ mice exhibit the hyperproliferation of endothelial cells, suggesting that Antxr1 negatively regulates the proliferation of endothelial cells and angiogenesis [8]. However, *Antxr1* expression is highly upregulated in the tumor endothelium, and the growth of human tumor xenografts, including melanoma, breast, colon, and lung cancer, is impaired in *Antxr1*^–/–^ mice. Moreover, an antibody against Antxr1 inhibits tumor-induced angiogenesis, suggesting that Antxr1 expression in the endothelium is required for the proliferation of endothelial cells and angiogenesis in cancer growth [30]. Therefore, the function of Antxr1 in the proliferation of endothelial cells differs between physiological conditions and cancer angiogenesis. Furthermore, knockdown of *Antxr1* reduced the proliferation of osteosarcoma and XWLC05 lung adenocarcinoma cells [31,32]. Thus, Antxr1 positively regulates the proliferation of both endothelial cells in cancer and the cancer cells themselves but negatively regulates endothelial cell proliferation in physiological conditions. Our study demonstrated that Antxr1 is a physiologically positive regulator of chondrocyte proliferation. Although *Antxr1* knockdown was reported to reduce Ccnd1 and to increase Cdkn1a and Cdkn1b expression in osteosarcoma cells [32], their expression was not changed in the limb cartilage of *Antxr1* tg mice compared with that in wild-type mice at E15.5 by microarray analysis (data not shown). Therefore, the mechanism of the positive regulation by Antxr1 in chondrocyte proliferation requires further investigation.

In the growth plate, terminal hypertrophic chondrocytes die by apoptosis and mineralization occurs due to their increased intracellular phosphate ions [26,27,28]. Chondrocyte hypertrophy and *Col10a1* expression are prerequisite for the subsequent apoptosis and mineralization, and cellular phosphate ion levels are closely related to chondrocyte maturation [28,33,34,35]. However, chondrocyte hypertrophy and *Col10a1* expression were not observed in the TUNEL-positive and mineralized regions, which were mainly observed in the resting chondrocyte layer, in *Antxr1* tg mice. This suggests that Antxr1 increased the amount of cellular phosphate ions, which induced apoptosis and mineralization without inducing chondrocyte maturation, or that other mechanisms, including the increase in intracellular calcium ions, are responsible for the apoptosis and mineralization. *Spp1* expression in the chondrocytes surrounding TUNEL-positive cells may have been induced by danger-associated molecular patterns (DAMPs) released from the necrotic cells, which originated from apoptotic chondrocytes, because *Spp1* expression is induced by inflammation [36,37]. We previously reported that overexpression of both *Ccnd1* and *Cdk6* increases BrdU uptake but induces chondrocyte apoptosis. Overexpression of both *Ccnd1* and *Cdk6* promotes G1/S cell cycle transition by phosphorylating pRB, but the cell cycle fails due to the dysregulation of E2F target genes [38,39]. Overexpression of *Antxr1* also likely promoted G1/S cell-cycle transition, but the cell cycle failed. In *Antxr1* tg mice, BrdU uptake and apoptosis were increased in both resting and proliferating layers but apoptosis was more prominent in the resting layer. Therefore, dysregulated promotion of the cell cycle may have played a role in apoptosis, but it was unlikely to be a major cause in *Antxr1* tg mice.

In conclusion, we demonstrated that Antxr1 positively regulates chondrocyte proliferation without affecting chondrocyte maturation, vascular invasion into the cartilage, or osteoblast differentiation. Furthermore, two-times overexpression of *Antxr1* in chondrocytes was sufficient to induce marked chondrocyte apoptosis, which was likely caused by mechanisms related to calcium or phosphate metabolism. Apoptosis is an important factor in osteoarthritis, and Runx2 is a causative molecule of osteoarthritis [36,40,41,42]. Thus, elucidation of the regulatory mechanisms for chondrocyte proliferation and apoptosis by Antxr1 will be beneficial not only for understanding physiological skeletal development but also for clarifying the pathogenesis of GAPO syndrome, osteoarthritis, and cancer growth.

## 4. Materials and Methods

### 4.1. Cell Culture, Adenoviral Transfer, Transfection of siRNA, and Real-Time RT-PCR Analysis

Primary chondrocytes were prepared from the limb skeletons of *Runx2*^–/–^ embryos at E18.5 or wild-type embryos at E15.5. Cells were plated on 48-well plates at a density of 7 × 10^4^/well in DMEM/Nutrient Mixture F-12 Ham (Sigma Aldrich, St. Louis, MO, USA) containing 5% fetal bovine serum (FBS) (Sigma Aldrich) and 10 μg/mL of human transferrin (Roche Diagnostics, Mannheim, Germany). The next day, cells were infected with an adenovirus expressing GFP or type II *Runx2*-GFP at a multiplicity of infection of 10 for 2 h. A total of 2 × 10^5^ cells was subjected to electroporation of 10 pmol of siRNA for the control or *Runx2* using the Neon Transfection System (Invitrogen, Carlsbad, CA, USA). RNA was extracted using ISOGEN (Wako, Osaka, Japan). Primary osteoblasts were prepared from calvariae of newborn mice as described previously [11]. RNA was extracted using ISOGEN (Wako). Real-time RT-PCR was performed using a THUNDERBIRD SYBR qPCR Mix (Toyobo, Osaka, Japan) and Light Cycler 480 real-time PCR system (Roche Diagnostics). We normalized the values obtained to those of β-actin. Primer sequences are shown in Supplementary Table S1.

### 4.2. Screening for Enhancers around the Antxr1 Region

To screen enhancers around the *Antxr1* region, we used datasets of ChIP-seq for H3K4me2, H3K4me3, H3K27ac, and p300, which we previously obtained for mouse primary rib chondrocytes [25]. ChIP-seq peaks were visualized in the CisGenome browser [43].

### 4.3. Reporter and ChIP Assays

The *Antrx1* 0.85-kb enhancer and 1.0-kb promoter (−762 to +309) were PCR amplified from mouse genomic DNA. The 0.85-kb enhancer was cloned into the NheI-XhoI site of the pGL4.23-Basic luciferase reporter vector containing a minimal promoter (Promega, Madison, WI, USA). The 1.0-kb promoter was cloned into the NheI-XhoI site of the pGL4.10-Basic luciferase reporter vector (Promega). To construct the luciferase reporter vector containing both the enhancer and promoter, the 0.85-kb enhancer was inserted into the BamHI site of pGL4.10 containing the 1.0-kb promoter. The recombinant constructs were confirmed by sequencing. A chondrogenic cell line, ATDC5, was purchased from the RIKEN Cell Bank (Tsukuba Science City, Japan). ATDC5 cells were seeded on 24-well plates at a density of 1.9 × 10^4^ cells/cm^2^ and cultured in DMEM/Nutrient Mixture F-12 Ham (Sigma Aldrich) containing 5% FBS (Sigma Aldrich) and 10 μg/mL of human transferrin (Roche Diagnostics) overnight. The cells were transiently transfected with a DNA mixture containing either the 1.0-kb promoter-luc, 0.85-kb enhancer-luc, or promoter/enhancer-luc and pRL-TK (Promega) with the empty vector (pSG5) or *Runx2*-expressing vector (pSG5-*Runx2*) using X-tremeGENE9 (Roche Diagnostics). Transfected cells were cultured for 48 hrs prior to harvest. Cell lysates were assayed for Firefly and Renilla luciferase activity using the Dual-Luciferase Reporter Assay System (Promega) according to the manufacturer’s instructions. Luciferase activity was normalized to Renilla luciferase activity. To induce chondrogenesis, ATDC5 cells were cultured in the above medium supplemented with 3 × 10^–8^ M sodium selenite (Sigma Aldrich) and 10 μg/mL of human recombinant insulin (Wako) for 15 days and were subjected to chromatin preparation. ChIP was performed according to the method previously described [44,45]. Immunoprecipitation was carried out overnight using Dynabeads M-280 (Invitrogen) and the following antibodies: Runx2 (F2) (Santa Cruz, Dallas, TX) or mouse IgG (Cell Signaling, Danvers, MA). Primer sequences are shown in Supplementary Table S1.

### 4.4. Generation of Antxr1^–/–^ Mice and Antxr1 tg Mice

*Antxr1*^–/–^ mice were generated as described previously [7]. The mice were backcrossed with C57BL/6 mice more than 7 times before analysis. The embryos at E15.5 and E16.5, newborns, female mice at 6 weeks of age, and male and female mice at 10 weeks of age were analyzed. To generate *Antxr1* tg mice, a 1902-bp mouse *Antxr1* cDNA fragment was cloned into the NotI site of a pNASSβ expression vector (Clontech, Mountain View, CA, USA), which contained the promoter and enhancer of the mouse *Col2a1* gene [18]. The construct was injected into the pronuclei of fertilized eggs from F1 hybrid mice (C57BL/6 × C3H). Mutant embryos were identified by genomic PCR. Transgene expression was measured by real-time RT-PCR using RNA purified from the vertebrae, ribs, or limbs. We normalized the values obtained to those of β-actin. We obtained 67 and 199 F_0_ embryos at E15.5 and E18.5, respectively; 14 embryos at E15.5 and 30 embryos at E18.5 were *Antxr1* tg; and we analyzed the F_0_ embryos. Prior to the study, all experimental protocols were reviewed and approved by the Animal Care and Use Committee of Nagasaki University Graduate School of Biomedical Sciences (No. 1903131520-2). Animals were housed 3 per cage in a pathogen-free environment on a 12-h light cycle at 22 ± 2 °C with standard chow (CLEA Japan, Tokyo, Japan) and had free access to tap water.

### 4.5. Skeletal, Histological, and Micro-CT Analyses

For skeletal preparations, embryos at E15.5 and E18.5 and newborn mice whose skin and internal organs were removed were fixed in 99% ethanol for 4 days. The skeletons were then stained for 38 hrs with 0.015% Alcian blue 8GX in 20% acetic acid and 80% ethanol at 37 °C with shaking. After washing with 99% ethanol overnight, the skeletons were stained with 0.004% alizarin red-S in 1% KOH overnight. They were cleared in 1% KOH solution and immersed in 50% glycerol and finally to 100% glycerol. To measure the lengths of femurs and tibiae, they were individually separated from the other skeletons. For histological analyses, mice were fixed in 4% paraformaldehyde/0.1 M phosphate buffer and were embedded in paraffin. Sections of 4 or 7 μm in thickness were stained with H-E or von Kossa or double-stained with von Kossa and H-E. The lengths of femurs and tibiae were also measured using the longitudinal sections through the center of the bones with the same anterior-posterior axis. TUNEL staining was performed using the ApopTag^®^ Peroxidase In Situ Apoptosis Detection Kit (Sigma Aldrich). To analyze BrdU incorporation, we subcutaneously injected BrdU into the back of pregnant mice at E18.5 at 100 μg/g of body weight 1 h before sacrifice and detected BrdU incorporation using a BrdU staining kit (Invitrogen). The sections were counterstained with hematoxylin. We carried out in situ hybridization using mouse *Col2a1*, *Col10a1, Spp1,* and *Col1a1* antisense probes as described previously [16]. In situ hybridization using sense probes produced no significant signals (data not shown). For ALP staining and double-staining with Alcian blue and von Kossa, E18.5 embryos were fixed in 4% paraformaldehyde at 4 °C for 2 h, washed with PBS at 4 °C for 1 hr, immersed in 20% sucrose at 4 °C overnight, embedded in O. C. T. Compound (Sakura Finetek, Tokyo, Japan), frozen in a refrigerated installation (Rikakikai UT-2000F, Tokyo, Japan) containing −100 °C hexane and pentane (10:3), and sectioned at 7-μm thickness using a Leica CM3050S (Leica Biosystems, Tokyo, Japan). For ALP staining, the cryosections were stained with the solution containing 0.1 mg/mL of Naphthol AS–MX phosphate (Sigma Aldrich), 0.05% *N*,*N*–dimethylformamide (Wako), 0.1 M Tris–HCl (pH: 8.5), and 0.6 mg/mL of Fast BB salt (Sigma Aldrich). For double-staining of Alcian blue and von Kossa, the cryosections were stained with Alcian blue and then stained with 5% silver nitrate in DDW. The skull length was measured using a micro-CT system (R_mCT, Rigaku Corporation, Tokyo, Japan). All measurements of the lengths of bones or skulls using skeletal preparations, histological sections, raw bones, or micro-CT images were done by two researchers in a double-blind manner.

### 4.6. Statistical Analysis

Values are shown as the mean ± SD. Statistical analyses were performed by the Student’s *t*-test. A *p*-value of less than 0.05 was considered significant.

## Supplementary Materials

Supplementary materials can be found in additional file. Supplementary materials can be found at https://www.mdpi.com/1422-0067/21/7/2425/s1.

## References

[1] Croix B.S., Bourne J. (2000). Genes Expressed in Human Tumor Endothelium. Science.

[2] Bradley K.A., Mogridge J., Mourez M., Collier R.J., Young J.A.T. (2001). Identification of the cellular receptor for anthrax toxin. Nature.

[3] Stranecky V., Hoischen A., Hartmannova H., Zaki M., Chaudhary A., Zudaire E., Noskova L., Baresova V., Pristoupilova A., Hodaňová K. (2013). Mutations in ANTXR1 Cause GAPO Syndrome. Am. J. Hum. Genet..

[4] Wajntal A., Koiffmann C.P., Mendonca B., Sotto M.N., Rati P.B.M., Opitz J.M., Epps-Quaglia D. (1990). GAPO syndrome (McKusick 23074)—A connective tissue disorder: Report on two affected sibs and on the pathologic findings in the older. Am. J. Med Genet..

[5] Nanda A., Carson-Walter E.B., Seaman S., Barber T.D., Stampfl J., Singh S., Vogelstein B., Kinzler K.W., Croix B.S. (2004). TEM8 Interacts with the Cleaved C5 Domain of Collagen α3(VI). Cancer Res..

[6] Werner E., Kowalczyk A.P., Faundez V. (2006). Anthrax toxin receptor 1/tumor endothelium marker 8 mediates cell spreading by coupling extracellular ligands to the actin cytoskeleton. J. Biol. Chem..

[7] Cullen M., Seaman S., Chaudhary A., Yang M.Y., Hilton M.B., Logsdon D., Haines D.C., Tessarollo L., Croix B.S. (2009). Host-derived tumor endothelial marker 8 promotes the growth of melanoma. Cancer Res..

[8] Besschetnova T.Y., Ichimura T., Katebi N., Croix B.S., Bonventre J.V., Olsen B.R. (2015). Regulatory mechanisms of anthrax toxin receptor 1-dependent vascular and connective tissue homeostasis. Matrix Boil..

[9] Komori T. (2020). Molecular Mechanism of Runx2-Dependent Bone Development. Mol Cells.

[10] Kawane T., Qin X., Jiang Q., Miyazaki T., Komori H., Yoshida C.A., Matsuura-Kawata V.K.D.S., Sakane C., Matsuo Y., Nagai K. (2018). Runx2 is required for the proliferation of osteoblast progenitors and induces proliferation by regulating Fgfr2 and Fgfr3. Sci. Rep..

[11] Qin X., Jiang Q., Miyazaki T., Komori T. (2018). Runx2 regulates cranial suture closure by inducing hedgehog, Fgf, Wnt and Pthlh signaling pathway gene expressions in suture mesenchymal cells. Hum. Mol. Genet..

[12] Nakashima K., Zhou X., Kunkel G.R., Zhang Z., Deng J.M., Behringer R.R., De Crombrugghe B. (2002). The novel zinc finger-containing transcription factor osterix is required for osteoblast differentiation and bone formation. Cell.

[13] Yoshida C.A., Komori H., Maruyama Z., Miyazaki T., Kawasaki K., Furuichi T., Fukuyama R., Mori M., Yamana K., Nakamura K. (2012). SP7 Inhibits Osteoblast Differentiation at a Late Stage in Mice. PLOS ONE.

[14] Nakatomi C., Nakatomi M., Matsubara T., Komori T., Doi-Inoue T., Ishimaru N., Weih F., Iwamoto T., Matsuda M., Kokabu S. (2019). Constitutive activation of the alternative NF-kappaB pathway disturbs endochondral ossification. Bone.

[15] Moriishi T., Ozasa R., Ishimoto T., Nakano T., Hasegawa T., Miyazaki T., Liu W., Fukuyama R., Wang Y., Komori H. (2020). Osteocalcin is necessary for the alignment of apatite crystallites, but not glucose metabolism, testosterone synthesis, or muscle mass. PLoS Genet..

[16] Inada M., Yasui T., Nomura S., Miyake S., Deguchi K., Himeno M., Sato M., Yamagiwa H., Kimura T., Yasui N. (1999). Maturational disturbance of chondrocytes inCbfa1-deficient mice. Dev. Dyn..

[17] Kim I.S., Otto F., Zabel B., Mundlos S. (1999). Regulation of chondrocyte differentiation by Cbfa1. Mech. Dev..

[18] Ueta C., Iwamoto M., Kanatani N., Yoshida C., Liu Y., Enomoto-Iwamoto M., Ohmori T., Enomoto H., Nakata K., Takada K. (2001). Skeletal Malformations Caused by Overexpression of Cbfa1 or Its Dominant Negative Form in Chondrocytes. J. Cell Boil..

[19] Takeda S., Bonnamy J.-P., Owen M.J., Ducy P., Karsenty G. (2001). Continuous expression of Cbfa1 in nonhypertrophic chondrocytes uncovers its ability to induce hypertrophic chondrocyte differentiation and partially rescues Cbfa1-deficient mice. Genome Res..

[20] Enomoto H., Enomoto-Iwamoto M., Iwamoto M., Nomura S., Himeno M., Kitamura Y., Kishimoto T., Komori T. (2000). Cbfa1 Is a Positive Regulatory Factor in Chondrocyte Maturation. J. Boil. Chem..

[21] Yoshida C.A., Yamamoto H., Fujita T., Furuichi T., Ito K., Inoue K.-I., Yamana K., Zanma A., Takada K., Ito Y. (2004). Runx2 and Runx3 are essential for chondrocyte maturation, and Runx2 regulates limb growth through induction of Indian hedgehog. Genome Res..

[22] St-Jacques B., Hammerschmidt M., McMahon A.P. (1999). Indian hedgehog signaling regulates proliferation and differentiation of chondrocytes and is essential for bone formation. Genome Res..

[23] Vortkamp A., Lee K., Lanske B., Segre G.V., Kronenberg H.M., Tabin C.J. (1996). Regulation of Rate of Cartilage Differentiation by Indian Hedgehog and PTH-Related Protein. Sci..

[24] Iwamoto M., Kitagaki J., Tamamura Y., Gentili C., Koyama E., Enomoto H., Komori T., Pacifici M., Enomoto-Iwamoto M. (2003). Runx2 expression and action in chondrocytes are regulated by retinoid signaling and parathyroid hormone-related peptide (PTHrP). Osteoarthr. Cartil..

[25] Ohba S., He X., Hojo H., McMahon A.P. (2015). Distinct Transcriptional Programs Underlie Sox9 Regulation of the Mammalian Chondrocyte. Cell Rep..

[26] Wu L.N.Y., Ishikawa Y., Sauer G.R., Genge B.R., Mwale F., Mishima H., Wuthier R.E. (1995). Morphological and biochemical characterization of mineralizing primary cultures of avian growth plate chondrocytes: Evidence for cellular processing of Ca2+ and Pi prior to matrix mineralization. J. Cell. Biochem..

[27] Mansfield K., Teixeira C., Adams C.S., Shapiro I. (2001). Phosphate ions mediate chondrocyte apoptosis through a plasma membrane transporter mechanism. Bone.

[28] Magne D., Bluteau G., Faucheux C., Palmer G., Vignes-Colombeix C., Pilet P., Rouillon T., Caverzasio J., Weiss P., Daculsi G. (2003). Phosphate is a specific signal for ATDC5 chondrocyte maturation and apoptosis-associated mineralization: possible implication of apoptosis in the regulation of endochondral ossification. J. Bone Miner. Res..

[29] Olsen B.R., Berendsen A.D., Besschetnova T.Y., Duan X., Hu K. (2016). Regulatory mechanisms of skeletal and connective tissue development and homeostasis – lessons from studies of human disorders. Int. J. Exp. Pathol..

[30] Chaudhary A., Hilton M.B., Seaman S., Haines D.C., Stevenson S., Lemotte P.K., Tschantz W.R., Zhang X.M., Saha S., Fleming T. (2012). TEM8/ANTXR1 blockade inhibits pathological angiogenesis and potentiates tumoricidal responses against multiple cancer types. Cancer Cell.

[31] Gong Q., Liu C., Wang C., Zhuang L., Zhang L., Wang X. (2017). Effect of silencing TEM8 gene on proliferation, apoptosis, migration and invasion of XWLC-05 lung cancer cells. Mol. Med. Rep..

[32] Cao C., Wang Z., Huang L., Bai L., Wang Y., Liang Y., Dou C., Wang L. (2016). Down-regulation of tumor endothelial marker 8 suppresses cell proliferation mediated by ERK1/2 activity. Sci. Rep..

[33] Wang D., Canaff L., Davidson D., Corluka A., Liu H., Hendy G., Henderson J.E. (2001). Alterations in the Sensing and Transport of Phosphate and Calcium by Differentiating Chondrocytes. J. Boil. Chem..

[34] Kakuta S., Golub E.E., Shapiro I.M. (1985). Morphochemical analysis of phosphorus pools in calcifying cartilage. Calcif. Tissue Int..

[35] Mwale F., Chetina E.V., Wu C.W., Poole A.R. (2002). The Assembly and Remodeling of the Extracellular Matrix in the Growth Plate in Relationship to Mineral Deposition and Cellular Hypertrophy: An In Situ Study of Collagens II and IX and Proteoglycan. J. Bone Miner. Res..

[36] Komori T. (2016). Cell Death in Chondrocytes, Osteoblasts, and Osteocytes. Int. J. Mol. Sci..

[37] O’Regan A., Berman J.S. (2000). Osteopontin: a key cytokine in cell-mediated and granulomatous inflammation. Int. J. Exp. Pathol..

[38] Ito K., Maruyama Z., Sakai A., Izumi S., Moriishi T., A Yoshida C., Miyazaki T., Komori H., Takada K., Kawaguchi H. (2013). Overexpression of Cdk6 and Ccnd1 in chondrocytes inhibited chondrocyte maturation and caused p53-dependent apoptosis without enhancing proliferation. Oncogene.

[39] Komori T. (2013). Regulation of Rb family proteins by Cdk6/Ccnd1 in growth plates. Cell Cycle.

[40] Kamekura S., Kawasaki Y., Hoshi K., Shimoaka T., Chikuda H., Maruyama Z., Komori T., Sato S., Takeda S., Karsenty G. (2006). Contribution of runt-related transcription factor 2 to the pathogenesis of osteoarthritis in mice after induction of knee joint instability. Arthritis Rheum..

[41] Liao L., Zhang S., Gu J., Takarada T., Yoneda Y., Huang J., Zhao L., Oh C., Li J., Wang B. (2017). Deletion of Runx2 in Articular Chondrocytes Decelerates the Progression of DMM-Induced Osteoarthritis in Adult Mice. Sci. Rep..

[42] Catheline S., Hoak D., Chang M., Ketz J.P., Hilton M.J., Zuscik M.J., Jonason J.H. (2019). Chondrocyte-Specific RUNX2 Overexpression Accelerates Post-traumatic Osteoarthritis Progression in Adult Mice. J. Bone Miner. Res..

[43] Ji H., Jiang H., Ma W., Johnson D.S., Myers R.M., Wong W. (2008). An integrated software system for analyzing ChIP-chip and ChIP-seq data. Nat. Biotechnol..

[44] Odom D.T., Zizlsperger N., Gordon D.B., Bell G.W., Rinaldi N., Murray H.L., Volkert T.L., Schreiber J., Rolfe P.A., Gifford D.K. (2004). Control of Pancreas and Liver Gene Expression by HNF Transcription Factors. Sci..

[45] Vokes S.A., Ji H., McCuine S., Tenzen T., Giles S., Zhong S., Longabaugh W.J., Davidson E.H., Wong W., McMahon A.P. (2007). Genomic characterization of Gli-activator targets in sonic hedgehog-mediated neural patterning. Dev..

