# Supplementary material for: Antxr1, Which is a Target of Runx2, Regulates Chondrocyte Proliferation and Apoptosis
Source: Int J Mol Sci. 2020 Mar 31;21(7):2425. doi: 10.3390/ijms21072425 (PMC7178079; doi:10.3390/ijms21072425)
Supplement: Supplementary file 1 [file ijms-21-02425-s001.pdf]

Supplementary Fig. 1

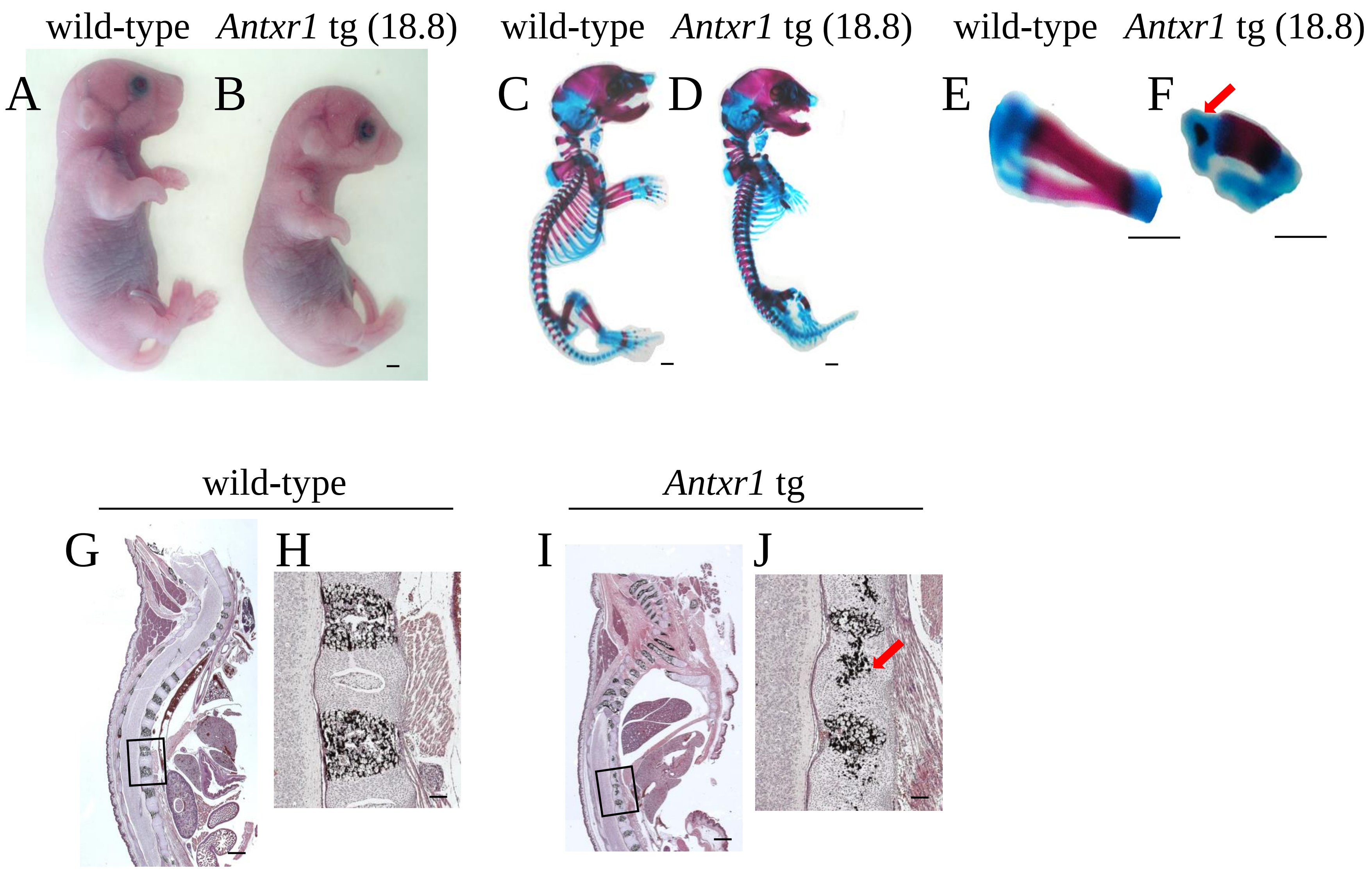

Supplementary Fig. 1 Appearance, skeletal system, and histological analysis of wild-type and *Antxr1* tg F_0 embryos with high transgene expression at E18.5

(A-F) Appearance (A, B), whole skeletal preparation (C, D), and magnified view of the tibiae and fibulae (E, F) of wild-type (A, C, E) embryo and *Antxr1* tg (B, D, F) F_0 embryo with high transgene expression. *Antxr1* expression in the tg embryo was 18.8-times higher than that in wild-type embryos. The arrow in F indicates ectopic mineralization in the cartilage in tibia. (G-J) Double-staining with H-E and von Kossa using sagittal sections of the trunk from wild-type (G) and *Antxr1* tg (I) F_0 embryos. The boxed regions in G and I are magnified in H and J, respectively. The arrow in J indicates ectopic mineralization in vertebral body. Scale bars: 1 mm (A-F, G, I), 100 µm (H, J).

Supplementary Table 1
Primer sequences for real-time RT-PCR and CHIP

real-time RT-PCR

	Forward	Reverse
	5'---------------------3'	5'---------------------3'
β-actin	CCACCCGCGAGCACAGCTTC	TTGTCGACGACCAGCGCAGC
Runx2	CCGCACGACAACCGCACCAT	CGCTCCGGCCCACAAATCTC
Antxr1	TACGGAGGATTCGACCTCTA	TGTTCCCTGTCCTCAGTTAG

CHIP

	5'---------------------3'
0.85-kb enhancer F1	ACAAGGCTTTCCTCTTGAGGGTC
0.85-kb enhancer R1	CTTGTGGGAAATCAGCAGGG
0.85-kb enhancer F2	AGAGTCATGCTTACCGCAGG
0.85-kb enhancer R2	CATACGCTCAGCTCCACCAA
